# Factors Associated with Periodontitis in Patients with and without HIV

**DOI:** 10.1155/2023/9929835

**Published:** 2023-04-29

**Authors:** Luanderson Lopes Pereira, Daniele Veiga Siqueira Amorim, Willian Brito Sampaio, Thais Almeida Cruz Azevêdo, Vanessa Bispo Pereira Cardoso, Felipe Barreto Lemos, Andressa Silva Chang, Fernanda Machado, Fernanda Pereira Lima, Frederico Sampaio Neves, Andreia Cristina Leal Figueiredo

**Affiliations:** ^1^Postgraduate Program in Dentistry and Health, Federal University of Bahia (UFBA), Salvador, Brazil; ^2^Department of Pediatric Oncology, Barretos Cancer Hospital, São Paulo, Brazil; ^3^Federal University of Bahia (UFBA), Salvador, Brazil; ^4^Department of Dental Radiology, Federal University of Bahia (UFBA), Salvador, Brazil; ^5^Department of Public Health, Federal University of Bahia (UFBA), Salvador, Brazil

## Abstract

**Purpose:**

The aim of this study was to compare clinical periodontal conditions in HIV-positive people on HAART with an HIV-negative group, in addition to investigating factors associated with periodontitis in the entire sample.

**Methods:**

This was a cross-sectional study. Data were collected by oral clinical examination for the diagnosis of periodontitis, review of medical records, and application of a questionnaire containing personal data, deleterious habits, and oral hygiene habits for the other variables. The results were analyzed by Pearson's *χ*^2^ test and Student's *t*-test. A logistic regression model was constructed for the multivariate analysis and periodontitis was defined as a dependent variable. The analysis was performed on the entire sample (HIV+ and HIV−) and also on the group consisting of only people living with HIV.

**Results:**

Individuals older than 43 years old and with HIV were more likely to develop moderate and severe periodontitis (47.80 and 4.84 times, respectively). When analyzing only HIV+, in addition to age (OR = 2.795; CI = 1.080−7.233), the use of nonnucleoside reverse transcriptase inhibitors (NNRTIs) (OR = 2.841; CI = 1.135−7.112) was also associated with moderate and severe periodontitis.

**Conclusion:**

It was possible to observe a higher prevalence of periodontitis among individuals with HIV, showing an association between the virus, advanced age, and moderate or severe periodontitis.

## 1. Introduction

Despite recent advances in HIV therapy, HIV infection still remains a global health problem. Among the most modern treatments is the highly active antiretroviral therapy (HAART), a medication regimen that has significantly changed the course of HIV disease into a manageable chronic illness demonstrating improved survival of infected individuals as a result of decreased viral load and increased CD4+ T lymphocyte count, which end up reducing the incidence of opportunistic infections and diseases related to immunosuppression [1]. Regarding oral diseases, the use of antiretroviral medications has proven to be very effective in reducing some manifestations caused by the virus, such as oral candidiasis, Kaposi's sarcoma, and atypical periodontal diseases (necrotizing gingivitis, necrotizing periodontitis) [2].

However, the reduction in the prevalence of chronic periodontitis in patients with HIV on HAART with controlled levels of T-cells still remains doubtful [3, 4]. Most works that investigate periodontitis in people living with HIV (PLWH) after the evolution of antiretroviral therapy report a similar prevalence between individuals with and without the virus. However, the number of works is small, mainly with an expressive and well-designed sample [3, 5–9].

Despite the benefits of antiretroviral therapy, the treatment does not completely restore the immune system and has been associated with adverse effects such as increased markers of premature aging [10, 11], residual chronic inflammation [12, 13], bone mineral loss [14], and oral and intestinal dysbiosis [15, 16]. Nonetheless, in the context of HAART, it appears that there has been a change in the profile of HIV-related diseases, and the presence of the virus is now associated with the exacerbation of chronic inflammatory diseases and disorders linked to aging [17].

Periodontitis is also a chronic disease mediated by immunoinflammatory factors and associated with older age [18]. In the early stages of periodontal disease, oral bacteria interact with periodontal tissue cells and trigger an inflammatory response that leads to destruction of the periodontium induced by the activation of lytic enzymes, mainly transglutaminases. The chronicity of the process, characterized by the constant activation of these enzymes, makes the treatment of periodontitis and tissue regeneration difficult [15, 19].

Some authors believe that periodontitis may influence worse HIV management due to overlapping immune activation caused by HIV and chronic periodontitis simultaneously, thus increasing the systemic inflammatory state and compromising treatment [17, 20, 21]. In addition, the inflamed gingival tissue can act as an HIV reservoir, facilitating the reactivation of the virus, being considered an obstacle to the eradication and control of HIV [5, 17, 22].

Periodontitis also demonstrates an intimate relationship with other systemic diseases, especially with regard to immune and inflammatory defenses. Among these diseases are diabetes [23], cardiovascular diseases [24], rheumatoid arthritis [25], and metabolic syndrome [26]. This is an important factor to be considered, since many chronic inflammatory conditions can interact with each other, potentiating the pathological effects of the diseases involved in this process [27].

Recent studies point to the potential of periodontitis, even in its early stages, to increase serum and salivary concentrations of NLRP3, an important inflammatory marker that determines the activation of IL-1*β* and its proinflammatory properties that include the recruitment of neutrophils and other cells' innate immune systems [28]. Elevation of this biomarker has also been indicated as a complicating factor involved in the process of controlling chronic diseases [29] such as diabetes [30], cardiovascular diseases [31], and HIV [32, 33].

It is of fundamental importance to constantly investigate the risk factors and prevalence of periodontitis among individuals with and without HIV in order to analyze the possible deleterious effects of the virus in influencing chronic periodontitis. Periodontitis is a disease that must be prevented and controlled, especially in people living with HIV, due to its potential for interaction with the virus, making the clinical control of the disease difficult [17, 22]. In addition, the identification and confirmation of possible risk factors linked to periodontitis allow the creation of public and private health strategies aimed at reducing the impact of the disease on vulnerable populations [34].

For the above reasons, the aim of this study was to compare clinical periodontal conditions in HIV-positive people on HAART with an HIV-negative group, in addition to investigating factors associated with periodontitis in the entire sample, including several variables that may be linked to disease progression. Our hypothesis is that HIV, regardless of other variables, may be associated with periodontitis.

## 2. Methods and Materials

### 2.1. Study Design

In this work, we applied the STROBE checklist to improve its quality [35]. This is a cross-sectional study carried out with participants (HIV+ and HIV−) randomly selected in the period from 2018 to 2020 from the Specialized Center for Diagnosis, Assistance and Research (CEDAP) located in Bahia state, Brazil. This Brazilian reference center provides free public health HIV-AIDS care services including HIV diagnostic tests. When the presence of the virus is confirmed, the reference center also offers the population antiretroviral medications and laboratory tests for HIV monitoring.

### 2.2. Ethical Aspects

This research was approved by the Research Ethics Committee of the Dentistry School of the Federal University of Bahia (protocol number 1.877.311) and was conducted in accordance with the Declaration of Helsinki [36] and its later amendments or comparable ethical standards [34].

### 2.3. Participants and Personal Data

The sample calculation was performed using the Epiinfo 3.5.1 statistical program and the following parameters: a confidence interval (CI) of 95%; a power of 80%; an expected frequency in the exposed group of 45%; an odds ratio (OR) of 2.45. The calculation indicated the need to include 180 participants. However, we decided to increase the sample size by 10% to take into account potential participant nonattendance, resulting in a sample of 200 individuals.

The sample consisted of 200 patients including HIV-exposed and HIV-unexposed people in a 1 : 1 ratio, of which 100 were individuals with a confirmed diagnosis of HIV/AIDS who attended the Specialized Center for Diagnostic, Treatment and Research (CEDAP) and the other 100 were healthy subjects who tested negative for HIV at the same care center. Serological testing was mandatory for all participants to avoid possible information bias.

Patient participation was voluntary and complied with the following inclusion criteria: individuals older than 18 years, patients submitted to a viral load test (ELISA and Western blot) to verify the presence of HIV, and individuals with at least 12 teeth in their dental arch. In addition, HIV+ patients needed to be on antiretroviral treatment, be under medical supervision, and have an undetectable viral load (<40 copies/mL).

The exclusion criteria were associated use of chemotherapy, individuals who reported having cardiovascular prostheses, diabetes, hypertension, osteoporosis, cardiovascular disease, pregnant women, history of necrotizing ulcerative periodontitis or necrotizing ulcerative gingivitis, and patients who underwent periodontal treatment or made use antibiotics less than 6 months before the periodontal examination, to avoid possible confounding factors between these characteristics and our main outcome–chronic periodontitis.

After checking the criteria, the individuals were invited to participate in the research. The participants were asked to sign the free and informed consent form. For those who accepted, the researchers applied a questionnaire to investigate their personal data: demographic data such as age (in years) and sex, deleterious habits (smoker and former smoker), presence of xerostomia (self-reported), and oral hygiene habits (daily tooth brushing, frequency of tooth brushing, and use of dental floss). After 7 days of patient recruitment, periodontal data were collected. There was no patient dropout and no loss of data involved in the final analysis.

### 2.4. Clinical Data

Data on HIV and antiretroviral therapy, such as start date, duration, combination therapy, modifications in the antiretroviral regimen, and if the treatment had already been interrupted or stopped for any reason, were obtained from the patients' records (SICLOM–Logistic Control System of Medicines). The system allows continuous updates in relation to the supply of medicines to patients on cART in various regions of the country, avoiding memory bias, since patients often do not know which type of medication is included in their therapeutic regimen and are not sure how long they have been using it.

As there is a wide variety of antiretroviral drugs, we analyzed the medications according to their type of mechanism of action: nucleoside reverse transcriptase inhibitor (NRTI), nonnucleoside reverse transcriptase inhibitor (NNRTI), protease inhibitor (PI), and integrase inhibitor (II).

Subsequently, some variables were created for the HIV+ group: CD4+ T-cell count (defined as the calculated average of all CD4+ T lymphocyte count tests for a given patient), NADIR CD4+ (the lowest historical CD4+ T-cell count for the referred patient), time with HIV (in months), and time on antiretroviral therapy (in months).

### 2.5. Periodontal Examination

Two examiners were trained and calibrated by the same researcher to perform the periodontal examination. For the calibration test, all teeth were probed and the probing depth and recession or hyperplasia were cataloged. Five participants were examined for calibration and probed twice by each examiner at an interval of 7 days.

Periodontal disease was evaluated through a clinical examination performed by a calibrated dental surgeon. The University of North Carolina millimeter probe (UNC-type) model PCP15 (Hu-Friedy®, Chicago, Illinois, USA) was used to assess the periodontal probing depths (PPD) and the distance between the cementoenamel junction and the gingival margin (CEJ-GM) at six sites per tooth in all dental units, except for the third molars due to the presence of false pockets that are common at the distal site and the fact that these teeth are increasingly rare in the oral cavity. Clinical attachment loss (AL) was calculated as the sum of the PPD and CEJ-GM measurements. Despite being hardworking and demanding longer time, we chose to examine all teeth in the oral cavity in order to reduce information bias and acquire an accurate diagnosis for each participant.

For the diagnosis of periodontitis in terms of severity, the CDC/AAP criteria proposed by Eke et al. [37] were used. The parameters used to classify periodontitis were ≥2 interproximal sites with AL ≥ 3 mm and ≥2 interproximal sites with PD ≥ 4 mm (not on the same tooth) or one site with PD ≥ 5 mm.

### 2.6. Data Analysis

For statistical analysis, periodontitis was categorized into two groups: group without periodontitis (participants who did not have periodontitis or had mild periodontitis) and group with periodontitis (participants with moderate or severe periodontitis). Periodontitis was defined as a categorical dependent variable.

Sociodemographic and pharmacological characteristics, deleterious habits, hygiene habits, xerostomia, and HIV were defined as independent variables. For the bivariate analysis and logistic regression, the quantitative variables were stratified according to their median, being categorized into two groups (above or below the median). Only the variables “CD4+ T lymphocyte count” and “NADIR CD4+” were stratified according to a cut-off point (350 and 200 cell/mm^3^) for being a borderline count to demonstrate immunosuppression of people living with HIV/AIDS.

**Figure 1:**
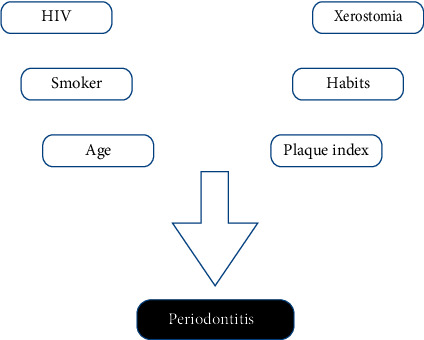
Theoretical analysis model.

The data were analyzed using SPSS 22.0. Pearson's *χ*^2^ test was used for the bivariate analysis. Means were compared using Student's *t*-test. For the multivariate analysis, logistic regression was performed using a theoretical model based on independent variables. Each block of variables was simultaneously compared with the outcome (periodontitis), allowing us to predict effects of one variable on another, as well as changes (Figure 1). To define which variables would be included in the logistic regression model, a bivariate analysis was conducted between the dependent (response) and independent variables separately. The variables that had a *P*-value lower than or equal to 0.20 remained in the final model, being identified as potential confounders. The other variables were considered effect modifiers and inserted into the final analysis. For the final model, a 95% CI and a *P*-value < 0.05 were established.

A multivariate analysis including only the HIV group was also performed, following the same parameters mentioned above. In this analysis, in addition to the variables already analyzed, specific variables of people living with HIV, such as time on antiretroviral medication, type of antiretroviral medication, and time with HIV and CD4 T-cell count, were also included.

## 3. Results

Intraexaminer agreement was tested and the correlation coefficient was 0.9328 (95% CI: 0.8348−0.9737).

A total of 200 subjects were examined, of which 100 had a diagnosis of HIV and the other 100 were not detected with the virus. Of the total number of participants, 121 (60.5%) were female, 81 (40.3%) had periodontitis, 73% brushed their teeth one to three times per day, and 52% reported using dental floss daily.

The distribution of sex, oral hygiene habits, and the presence of xerostomia and periodontitis in the groups with and without HIV can be seen in Table 1.

Among the participants without HIV, 66% were female and all of them reported brushing their teeth up to three times per day—even though 61% claimed not to floss every day. Regarding deleterious habits, 88% of the group were nonsmokers (of which 84% had never smoked and 16% were former smokers), 79% did not have xerostomia and 27% had moderate or severe periodontitis (Table 1). Moreover, this group had an average of 45.12% (SD = 21.43), of their dental surfaces with visible bacterial plaque and had 38.33% of sites with bleeding on probing (SD = 25.07) (Table 2).

Among the group of patients infected with HIV, 55% were female, 53% reported brushing their teeth more than four times per day, 65% used dental floss daily, 84% were smokers, 60.4% had xerostomia, and 54% had periodontitis (Table 1). Moreover, this group had a mean age of 41.03 years (SD = 9.59), a visible bacterial plaque index of 41.74% (SD = 19.52), and an average number of bleeding sites on probing of 40.67% (SD = 27.24) (Table 2).

Participants with HIV were living ∼ 90.27 months with the virus (SD = 65.16) and undergoing 77.17 months of antiretroviral therapy (SD = 56.33). Regarding the immunological profile, the participants had average CD4+ T-cell and NADIR CD4+ counts of 556.03 cells/mm^3^ (SD = 313.57) and 330.03 cells/mm^3^ (SD = 249.82), respectively (Table 3).

The bivariate analysis of the correlation between periodontitis and the independent variables (demographic characteristics, oral hygiene habits, deleterious habits, xerostomia, and HIV) is shown in Table 4. Individuals who were over 43 years old (OR = 1.557; CI = 0.882−2.747), brushed their teeth four times or more per day (OR = 2.208; CI = 1.670−4.177), were smokers (OR = 2.152; CI = 1.211−3.823) or former smokers (OR = 2.152; CI = 1.211−3.823), and were infected with HIV (OR = 3.064; CI = 1.698−5.529) were more likely to develop periodontitis. The other variables were not significantly associated with periodontitis (*p* > 0.05) (Table 4).

All variables that obtained statistical significance above the cut-off point (*p* < 0.20) were included in the final multivariate analysis model, while the other variables were considered as possible confounding factors. The results of the multivariate analysis are presented in Table 5, which shows the variables that remained in the final model. After adjustments based on the theoretical model, individuals older than 43 years (OR_A_ = 4.674; CI = 2.245−9.732) and infected with HIV (OR_A_ = 5.554; CI = 1.596−19.324) were more likely to develop periodontitis (Table 5).

The results of the multivariate analysis including only individuals with HIV and the presence of moderate and severe periodontitis as an outcome are shown in Table 6. It can be observed that among the participants with HIV, older individuals (OR = 2,795; CI = 1,080−7,233) and those making use of NNRTIs (OR = 2,841; CI = 1,135−7,112) were twice as likely to have moderate and severe periodontitis. Neither the influence of nucleotide reverse transcriptase inhibitors (NRTIs) nor whether their use would be associated with a greater chance of developing periodontitis was analyzed because 100% of our HIV+ sample took or had already taken this type of medication in their antiretroviral treatment regimen (Table 6).

## 4. Discussion

The main associated factors found in our sample were older age and presence of HIV, evidencing the strong influence of these variables on periodontitis (Table 5). Even after the inclusion of other variables that are strongly associated with periodontitis such as brushing habits, smoking habits, xerostomia, and plaque index in the multifactorial regression analysis, only age and the presence of HIV were associated with the presence of moderate and severe periodontitis after the model end of the analysis.

As already mentioned, periodontitis is a chronic inflammatory disease modulated by the immune system that can be associated with a series of risk factors, similar to those shared by aging. Additionally, chronic medication use, comorbidities, immunosenescence, and alteration of the inflammatory and immune responses resulting from aging can influence the onset and progression of periodontitis [38, 39]. In our sample, it was possible to observe a significant correlation between periodontitis and older age (OR_A_ = 4.674). These data corroborate other epidemiological studies that suggest an increase in the prevalence of periodontitis with advancement of age [40, 41].

People living with HIV have an increasing life expectancy, which increases exposure to age-related risk factors that can be potentiated by the deleterious factors of HIV infection [10, 12, 13]. It is also possible to observe an increase in the incidence or worsening of age-related chronic inflammatory diseases such as cardiovascular diseases [42], diabetes [43], hypertension [44], metabolic syndrome [45], tuberculosis [46], and possibly periodontitis [5, 47, 48].

Furthermore, studies have shown the influence of HIV and antiretroviral medication on accelerating the aging process in people living with HIV. The mechanisms by which HIV can influence accelerated immunosenescence in young individuals can be explained by chronic immune activation, high profile of inflammation played by B cells, replicative senescence of CD4 and CD8 T-cells, frequencies of naïve cells, and the innate immune response presented by infected individuals [39, 49].

Even individuals using antiretroviral therapy may have deleterious effects related to aging, such as mitochondrial dysfunction, loss of proteostasis, and exhaustion of stem cells or epigenetic changes [10, 33]. Some viral proteins such as Nef, Tat, or Vpr can induce T-cell apoptosis, interfere with autophagy, and promote cell aging. HIV infection itself causes depletion of mitochondrial DNA levels, which induces the production of reactive oxygen species and deregulates the methylome in several locations [13, 33, 39].

Regarding HIV, our analytical results revealed that infected individuals were five times more likely to have moderate and severe periodontitis than those without HIV (OR_A_ = 5.554) (Table 5). There are several factors that can influence the course of periodontitis in people living with HIV. First, the virus infection acts as a modifying factor in periodontal diseases, showing a close relationship with several inflammatory and opportunistic periodontal diseases. Second, it is frequently associated with the occurrence of acute periodontal diseases, linear gingival erythema, and exacerbation of pre-existing chronic periodontitis [5, 50].

One factor influencing the prevalence and severity of periodontitis in this population can be explained by the infection itself, which contributes to the destruction of the oral mucosal epithelium, consequently favoring microbial translocation and inducing a systemic inflammatory state [16]. The high viral replication and marked depletion of CD4+ T lymphocytes in these cells reduce the production of interleukin-17 (Th17) and interleukin-22 (Th22) cells, resulting in systemic immune activation and possible exacerbation of the periodontal [51–54].

The evaluation of CD4+ T lymphocyte count is an important tool to monitor the evolution of HIV, HIV-1-infected adults with low CD4+ T-cell count were shown to have twice the risk of clinical attachment loss and tissue breakdown than noninfected controls [55, 56]. Its numerical decrease and function alteration lead to a suppression of the immune response and an increase in oral opportunistic infections and other periodontal diseases. The profound suppression of immunity in individuals who do not use antiretroviral therapy seems to enhance the risk of the development of atypical periodontal diseases [2]. In contrat, the increase in T-cell count provided by HAART regulates the cytokine network and the amount of macrophages, leukocytes, and dendritic cells, making the organism more efficient in fighting infections, in addition to improving tissue repair and healing [57]

Although our entire HIV+ sample was on HAART and had a satisfactory median CD4 T-cell count of 556.03 (SD: 313.57), the HIV-infected individuals were more likely to have moderate and severe periodontitis than the uninfected ones, regardless of other variables that may also influence periodontitis, such as the presence of xerostomia, smoking, hygiene habits, socioeconomic status, diet, mental health, age, and microbiological factors [58, 59].

Despite the benefits of antiretroviral therapy, treatment does not fully restore the immune system and has been associated with adverse effects such as increased markers of premature aging [10, 11], residual chronic inflammation [12, 13], bone mineral loss [14], and oral and intestinal dysbiosis [15, 16], these changes could justify a possible explanation for the high rate of periodontitis in our sample with HIV. Unfortunately, we could not analyze inflammatory markers or oral microbiomes in our sample, due to the high cost of these techniques, especially when studying a large sample of 200 participants. The great bacterial diversity and complexity in the oral microbiota of HIV-infected individuals may be related to the progression and severity of chronic periodontitis, which can lead to an imbalance between microbial aggression and the host's immune response, enhancing the effects of periodontitis [5, 16, 17, 60].

Although the groups with and without HIV presented a very similar mean age and plaque index (Table 2), they showed a great difference in relation to the prevalence of moderate and severe periodontitis (54% in infected individuals against only 27% in uninfected ones) (Table 1). Despite being possibly directly linked to the presence of HIV, the higher prevalence of periodontitis in this population may also have been influenced by the effect of other characteristics associated with the onset and progression of periodontitis such as xerostomia or smoking. It is true that the HIV group was composed of a higher number of smokers and ex-smokers, individuals with xerostomia, and patients who used less dental floss, despite having reported a greater number of daily brushing, than the group without the virus (Table 1).

Concerning the habit of smoking, even though smoking was identified in the bivariate analysis as a risk factor associated with the presence of moderate and severe periodontitis (Table 4), when analyzing all variables together through logistic regression no correlation between smoking habit and periodontitis was found to be statistically significant. Although this result may be inherent to the specific characteristics of our sample, it must be highlighted that smoking is a risk factor for periodontal disease widely reported and studied in the scientific literature [58, 61]. The amount of cigarettes smoked by the participants and the time that the ex-smokers had not smoked were also not included in the present study. The inclusion of these characteristics would be important for the development of future studies since tobacco has cumulative deleterious effects that extend over the long term [62].

Microbiological studies indicate that the oral biofilm in smokers is composed of more periodontopathogenic bacteria compared with nonsmokers. Suggesting that smoking considerably affects the subgingival bacterial symbiosis by the microbial–host ecological interaction and consequently the severity of periodontitis [63, 64]. Furthermore, the harmful substances present in cigarettes and their by-products exert a vasoconstrictor effect not only on the peripheral circulation but also on the gingival circulation, thus reducing the functional activity of leukocytes and macrophages in the saliva, as well as the chemotaxis and phagocytosis of polymorphonuclear leukocytes [65, 66].

Although the global prevalence of smoking has been declining, the proportion of people living with HIV who are smokers remains high and is almost twice the number of smokers in the general population [67, 68]. This tendency was observed in our sample, where 84% of the HIV+ patients were smokers against only 22% of uninfected individuals. In an attempt to reduce these numbers, many studies have addressed approaches to minimize the damage caused by tobacco without necessarily requiring abstinence through the use of alternatives such as adhesives, gums, lozenges, mouth sprays, and transdermal products. Many HIV-positive smokers have difficulties in adopting these alternatives since smoking helps reduce the impacts of living with the disease-related stigma [61, 69].

Besides being essential for maintaining the integrity of the oral mucosa, the saliva exerts local immune functions against periodontal pathogens and increases patient comfort, favoring chewing, swallowing, digestion, speech, and quality of life [70, 71]. In the present study, it was possible to observe that 60% of the participants with HIV had xerostomia, while among the participants without the virus, only 20% presented this condition. Metabolic alterations caused by antiretroviral medication and HIV infection can significantly reduce salivary flow and negatively alter periodontal repair. In addition to quantitative changes, the medication can generate changes in salivary composition, mainly in the concentration of beta-defensins, biomarkers associated with periodontal disease in non-HIV-infected patients that can be found in greater amounts in patients on HAART [72]. In the present study, there was a significant difference in the number of people with xerostomia between the group with and without HIV (Table 1). However, when analyzing periodontitis as an outcome in our multivariate analysis, the presence or absence of xerostomia was not associated with moderate or severe periodontitis (Table 5).

It is well established in the literature that the accumulation of dental plaque on teeth leads to gingivitis, which in some cases can progress to chronic periodontitis if not treated early [15]. Even though our study showed that people with a higher plaque index are more likely to have periodontitis, these results were not statistically significant (OR = 1.445; *p* ≤ 0.241) (Table 5). The lack of correlation between plaque index and the presence of periodontitis may be an inherent characteristic of our sample or an issue of variable categorization.

Toothbrushing is an important habit for preventing oral diseases. Indeed, there is ample evidence that mechanical and chemical methods of plaque control can prevent gingivitis and periodontitis. Provided that cleaning is sufficiently thorough, increased by cleaning of the interdental regions with efficient devices, and performed at appropriate time intervals, it can reliably control plaque accumulation [15, 73]. The measurement of the amount of daily brushing of each patient in our study was self-reported, which could have produced an information bias since the participant may have omitted the true number of times they brush their teeth per day. In addition, we did not know how this brushing was performed, the interval between brushings, and how each individual performed the manual dexterity, which ended up limiting our understanding of the complete removal of microbial biofilm. As already reported, biofilm removal may be incomplete due to the physical and cognitive limitations of the patient during the oral cavity cleaning process [74].

Access to basic procedures, preventive and educative oral actions are essential to avoid the onset of periodontitis, especially in vulnerable populations such as people living with HIV (PLWH) [75]. In our sample no statistically significant relationship between barriers to accessing oral health services and presence of moderate and severe periodontitis (OR = 0.523; *p* ≤ 0.167) was found. There are many barriers that interfere with the promotion of universal access to PLWHA, such as socioeconomic inequalities, ethnic and gender disparities, availability of social resources, geographical barriers, cultural differences between health professionals, and the user and stigma fear or experience [75, 76]. Despite analyzing the presence of barriers, we did not include the type of barrier reported by patients, this should be a factor to be considered when analyzing the incidence and prevalence of diseases in vulnerable populations in future studies. Nevertheless, studies conducted in other countries suggest that PLWH have limited access to oral care, which can increase the incidence of periodontitis and aggravate oral diseases that could be treated early or even prevented with basic conducts [77, 78].

We collected and analyzed, separately, variables that assessed the use of antiretroviral medications, hygiene habits, and immunological and social factors of people living with HIV (PLWH) so as to understand how these characteristics were associated and distributed among the group. Apart from age, the only variable that was related to the presence of moderate and severe periodontitis and obtained statistical significance in the group with HIV was the “use of non-nucleotide reverse transcriptase inhibitors (NNRTIs)”. Individuals who used this class of antiretroviral medication were twice as likely to have periodontitis (OR = 2.841; CI = 1.135−7.112) (Table 6).

NNRTIs seem to potentiate or accelerate manifestations of chronic diseases, especially those linked to aging, in addition to being associated with reduced bone mass and hypovitaminosis D. In users of Efavirenz, a type of antiretroviral drug belonging to this drug class (NNRTI), a decrease in plasma levels of 25-OH vitamin D can be observed. The reduction in the amount of vitamin D may be an important factor that has been studied and associated with osteoporosis and periodontitis. When used for a long time, this class of medication can cause a number of adverse and unwanted factors in the patient's body [79, 80]. Its use has already been associated with the presence of oxidative stress, originating mainly within the mitochondria. This event characterizes the main source of production of oxygen species (ROS) in mammalian cells, which, in turn, accelerates the aging process [81–83]. Oxidative stress and increased production of oxygen species (ROS) are also involved in the progression of periodontitis, one can potentiate the other, since inflammation can trigger oxidative stress and oxidation can also induce inflammation [84, 85].

In addition to oxidative stress, NNRTIs can increase endothelial permeability by suppressing junctional proteins necessary for correct epithelial barrier functionality. The increase in capillary permeability is associated with the development of a series of diseases, especially inflammatory ones [86]. Several studies have reported the power of these drugs to reduce cell proliferation and compromise cell viability. The relationship between some NNRTI drugs and the proinflammatory state appears to be critical to understand the pathogenesis of various side effects [87–90].

Changes in bone metabolism and reduced bone mass have been related to HIV infection and the use of antiretroviral therapy, both in young and old adults, which could be linked to the higher prevalence of periodontitis in this population [91]. Both HIV infection and antiretroviral drugs used by people living with HIV/AIDS (PLWHA) can cause changes in bone metabolism, resulting in low bone density–and representing an important factor associated with periodontitis [5].

Other drug classes have also been associated with adverse events caused by antiretroviral medication. Tenofovir (TDF), belonging to the class of NRTIs, has been considered a harmful agent for bone mineralization, the change in the therapeutic scheme with the substitution for other antiretroviral drugs showed an improvement in the users' bone mineral density [92]. By altering osteoblast gene expression, it can lead to functional defects of these cells, decreasing bone formation [93]. Another mechanism linked to bone loss in Tenofovir users is vitamin D deficiency, which causes an increase in the serum concentration of PTH, and consequently, bone remodeling, leading to a reduction in bone density [94].

We could not analyze whether the use of TDF or other medication belonging to the NRTIs was related to periodontitis because our entire sample uses this type of medication in their therapeutic regimen (Table 6). Antiretroviral therapy usually consists of the combination of three or more types of drugs taken in association [95]. However, each drug can generate specific adverse effects and be related in a different way to the individual's metabolism, which makes it difficult to analyze the effect of each medication in vivo on human populations, as the medications are used in association and often undergo changes, depending on the therapeutic regimen proposed for each individual [10, 96]. Therefore, new research strategies should be used to try to measure the long-term action of each drug used by the virus carriers. Due to the complexity of factors related to the HIV therapy, this is a condition of difficult analysis, representing a real challenge for researchers and health professionals.

In the present study, it was possible to observe a higher prevalence of periodontitis among individuals with HIV, showing an association between the virus, advanced age, and moderate or severe periodontitis. The results emphasize the importance of oral health care for people living with HIV, especially the elderly infected with the virus. Even on antiretroviral treatment and with a satisfactory CD4 T-cell count, individuals with the virus were more likely to have the disease, indicating the need to create effective public policies aimed at this population.

Among HIV-infected patients, it was possible to observe that in addition to age, the use of NNRTIs was identified as a possible risk factor for periodontitis. It is clear that the use of antiretroviral drugs is a protective factor and can offer several benefits to HIV-infected individuals. However, some studies point to the side effects of these drugs as possible factors associated with disorders and diseases, especially bone disorders, which justifies the need for developing new research so as to evaluate the safety and efficacy of these medications.

## Figures and Tables

**Table 1:** Distribution of sex, oral hygiene habits, xerostomia, and periodontitis in the groups with and without HIV.

Variable	Without HIV		With HIV		Total
	*n*	%	*n*	%	*N*
Sex					
Female	66	66	55	55	121
Male	34	34	45	45	79

Frequency of daily tooth brushing					
1–3 times	100	100	47	47	147
4 or more times	0	0	53	53	53

Daily use of dental floss					
Yes	39	39	65	65	104
No	61	61	35	35	96

Smoker					
Yes	12	12	84	84	96
No	88	88	16	16	104

Former smoker					
Yes	16	16	75	75	81
No	84	84	25	25	29

Xerostomia					
Yes	20	20	60	60	80
No	80	80	40	40	120

Periodontitis					
Yes	27	27	54	54	81
No	73	73	46	46	119

**Table 2:** Distribution of means between groups with and without HIV virus according to age, plaque index, and bleeding on probing.

Variables	Without HIV		With HIV	
	Means	Standard deviation	Means	Standard deviation
Age	44.81	13.94	41.03	9.59
Plaque index	45.12	21.43	41.74	19.52
Bleeding on probing	38.33	25.07	40.67	27.24

**Table 3:** Distribution of means and medians according to clinical and immunological of participants with HIV.

Periodontitis			
	Means	Median	Standard deviation
Time with HIV (in months)	90.27	72.00	65.16
CD4+ T-cell count	556.03	525.05	313.57
NADIR CD4+	330.03	282.00	249.82
Time on antiretroviral therapy (in months)	77.17	72.00	56.33

**Table 4:** Bivariate analysis of the association between periodontitis and demographic characteristics, oral hygiene habits, and xerostomia in the groups with and without HIV.

Periodontitis					
	No (%)	Yes (%)	OR_B_	95% CI	*p*-value
Sex					0.227
Male	39.2	60.8	1.0	–	
Female	47.9	52.1	0.702	0.395−1.1247	

Plaque index					0.178^*∗*^
0−42.3%	49.5	50.5	1.0	–	
>42.3%	40.0	60.0	1.469	0.839−2.573	

Age					0.126^*∗*^
Up to 42 years	64.7	35.3	1.0	–	
>43 years	54.1	45.9	1.557	0.882−2.747	

Daily tooth brushing					0.014^*∗*^
1–3 times	54.4	45.6	1.0	–	
>4 times	17.0	83.0	2.208	1.670−4.177	

Daily use of dental floss					0.588
No	42.7	57.3	1.0	–	
Yes	46.2	53.8	2.152	1.211−3.823	

Smoker					0.009^*∗*^
No	57.7	42.3	1.0	–	
Yes	30.2	69.8	3.150	1.757−5.650	

Former smoker					0.002^*∗*^
No	54.6	45.4	1.0	–	
Yes	32.6	67.4	2.491	1.391−4.463	

Xerostomia					0.713
No	46.5	53.5	1.0	–	
Yes	42.7	57.3	1.168	0.649−2.100	

HIV infection					0.001^*∗*^
No	63.0	37.0	1.0	–	
Yes	26.0	74.0	4.846	2.650−8.863	

Pearson's *χ*^2^ test.  ^*∗*^Bold asterisk variables that go to multivariate analysis because they have *p* value < 0.200.

**Table 5:** Multivariate analysis of periodontitis and associated factors in the groups with and without HIV.

Periodontitis			
	OR_A_	95% CI	*p*-value
Plaque index			0.241
0−42.3%	1.0	–	
>42.3%	1.445	0.781−2.674	

Age			**0.000**
Up to 42 years	1.0	–	
>43 years	4.674	2.245−9.732	

HIV infection			**0.007**
No	1.0	–	
Yes	5.554	1.596−19.324	

Daily tooth brushing			0.156
1−3 times	1.0	–	
>4 times	0.491	0.184−1.313	

Smoker			0.780
No	1.0	–	
Yes	0.873	0.336−2.266	

Former smoker			0.638
No	1.0	–	
Yes	1.222	0.529−2.821	

Logistic regression. Bold variables with *p*-value ≤ 0.05.

**Table 6:** Bivariate analysis of the association between periodontitis and demographic characteristics, habits, serological and pharmacological variables in the group with HIV.

Periodontitis					
	No (%)	Yes (%)	OR_B_	95% CI	*p*-value
Age					0.031^*∗*^
Up to 41 years	35.3	64.7	1.0	–	
>42 years	16.3	83.7	2.795	1.080−7.233	

Daily tooth brushing					0.797
Up to three times	23.1	76.9	1.0	–	
>3 times	26.4	73.6	0.835	0.211−3.303	

Smoker					0.253
No	23.8	76.2	1.0	–	
Yes	37.5	62.5	1.920	0.620−5.943	

Former smoker					0.240
No	28.8	71.2	1.0	–	
Yes	16.7	83.3	0.495	0.151−1.623	

CD4 count					0.811
Up to 200 cell/mm^3^	22.2	77.8	1.0	–	
>200 cell/mm^3^	25.9	74.1	0.818	0.158−4.238	

CD4 count					0.945
Up to 350 cell/mm^3^	25.0	75.0	1.0	–	
>350 cell/mm^3^	25.7	74.3	0.963	0.331−2.802	

NADIR CD4					0.497
Up to 200 cell/mm^3^	30.0	70.0	1.0	–	
>200 cell/mm^3^	23.4	76.6	1.400	0.530−3.699	

Plaque index					0.065
Up to 45.0%	35.6	64.4	1.0	–	
>45.0%	18.2	81.8	2.483	0.932−6.612	

Time on antiretroviral therapy					0.951
Up to 72 months	25.5	74.5	1.0	–	
>72 months	26.1	73.9	971	0.384−2.460	

Time with HIV					0.211
Up to 72 months	31.4	68.6	1.0	–	
>72 months	20.4	79.6	1.783	0.716−4.440	

Used NNRTI's					0.023^*∗*^
No	38.5	61.5	1.0	–	
Yes	18.0	82.0	2.841	1.135−7.112	

Used PI's					0.955
Yes	25.8	74.2	1.0	–	
No	26.3	73.7	974	0.388−2.442	

Used II's					0.076
Yes	46.2	53.8	1.0	–	
No	23.0	77.0	2.871	0.865−9.527	

Used NRTI's					#
Yes	26.0	74.0			
No	0.0	0.0			

Barriers to accessing free public dental care					0.065
Yes	38.6	61.4	1.0	–	
No	54.8	45.3	520	0.260−0.040	

Pearson's *χ*^2^ test.  ^*∗*^Bold asterisk variables with *p*-value ≤ 0.05. ^#^No statistics were calculated because this variable is a constant, all individuals used NRTI's.

## Data Availability

The data used to support the findings of this study are available from the corresponding author upon request.
